# A systemic longitudinal case study of the eMed GP at hand digital first primary care model

**DOI:** 10.1007/s44250-026-00344-9

**Published:** 2026-02-05

**Authors:** Iqra Shahzad, Melanie King

**Affiliations:** The Wolfson School of Mechanical, Electrical and Manufacturing Engineering, Loughborough University, Loughborough, LE11 3TU UK

## Abstract

The shift toward digital-first healthcare models presents both opportunities and challenges for health systems worldwide. This case study critically examines the evolution of eMed GP at Hand (formerly Babylon GP at Hand) within the NHS, tracing its journey from an innovative digital provider to the largest GP practice in England, and ultimately, its downsizing. By bringing together an analysis of reviews, media coverage, and existing research, the study evaluates the model’s impact on accessibility, continuity of care, and health inequalities. Findings reveal that while the digital-first approach improved access for younger, healthier populations, it inadequately served vulnerable groups, such as the elderly and those with complex conditions. The study also highlights systemic challenges, such as limitations in Babylon’s business model, regulatory gaps in digital health oversight, and the complexities of integrating private sector innovation within public healthcare systems. These insights emphasise the necessity for robust regulation, tailored digital solutions, and a complementary relationship between digital and traditional care models to ensure sustainable and equitable healthcare delivery.

## Introduction

Digital-first healthcare models are reshaping the delivery of primary care by promising greater convenience and cost-effectiveness and represent a transformative shift in primary care delivery [1–3]. In the United Kingdom, the National Health Service (NHS) has embraced these innovations through frameworks such as the General Practice Forward View [4] and the NHS Long Term Plan [5], both of which highlight technology as a vehicle for modernising primary care. Despite their apparent advantages, these models also raise concerns around health equity, continuity of care, and integration within traditional healthcare systems [6, 7].

Within this evolving context, eMed GP at Hand (https://www.gpathand.nhs.uk/), formerly Babylon GP at Hand, stands out as one of the earliest large-scale implementers of a digital-first GP service. See Table 1 for a timeline of key events for GP at Hand [8, 9]. From its initial promise of on-demand, app-based consultations to its more recent scaling challenges, eMed GP at Hand presents a case that captures both the potential and pitfalls of digital healthcare. The service’s rapid rise and subsequent downsizing underscore the importance of critically examining how such models adapt—particularly in public systems like the NHS that must balance innovation with equitable care for diverse populations.

**Table 1:** Timeline of key events for Babylon/eMed GP at hand

Phase	Year	Date	Key Event(s)
**Launch & Expansion**	2017	November	⇒ Launch of Babylon GP at Hand ⇒ Registration for service begins
	2018	March	⇒ Early Expansion ⇒ Increased user registrations
**Regulatory & Reviews**		November	⇒ Regulatory Scrutiny ⇒ NHS discussions on appointments
	2019	June	⇒ IPSOS MORI Evaluation ⇒ Independent review commissioned
**Challenges & Growth**		December	⇒ Ongoing Controversies ⇒ CCG concerns on patient selection
	2020	March	⇒ Pandemic surge ⇒ Spike in remote consultations
	2021	February	⇒ Technical issues ⇒ Criticism over continuity of care
**Financial Struggles & Transition**	2022	October	⇒ Birmingham closure ⇒ Service withdrawal due to financial pressures
	2023	August	⇒ Administration ⇒ UK arm acquired by eMed
		September	⇒ Rebranding ⇒ Service renamed to eMed GP at Hand
**Future & Adaptation**	2024	Onwards	⇒ Ongoing adaptation ⇒ Service exclusively in London; future expansion unclear

Against this backdrop, the present study undertakes a longitudinal analysis of eMed GP at Hand from its launch in 2017 through to mid-2024. In doing so, it aims to address the following research questions:

1. How has eMed GP at Hand’s digital-first approach affected healthcare accessibility, especially for populations with complex needs, limited digital skills, or other vulnerabilities?
2. To what extent has the service maintained continuity of care, and how do patients perceive the trade-offs between convenience and sustained provider–patient relationships?
3. What systemic factors, such as funding models, regulatory oversight, and public–private collaboration, have facilitated or hindered eMed GP at Hand’s integration into the NHS, and what lessons can be drawn for future digital healthcare initiatives?

By exploring these questions through a case study methodology, the research provides an in-depth look at user experiences, service adaptations, and broader structural influences shaping digital healthcare’s trajectory. It builds on prior evaluations, including the Ipsos MORI review [9], and extends its scope by examining the service’s evolution over time. Ultimately, this study seeks to inform stakeholders about the conditions under which digital-first models can thrive in a manner that remains both sustainable and equitable.

## Methodology

### Research design and rationale

A case study approach was chosen to explore the evolution of eMed GP at Hand over an extended period, enabling an in-depth examination of user perceptions, service delivery, and organizational changes [10]. Given the complexity of digital healthcare models, case studies provide a rich, contextual understanding that is particularly valuable for capturing the nuances of health service innovation.

Longitudinal data collection spanning from December 2017 to June 2024 was critical for capturing how user satisfaction, media coverage, and policy discourse shifted throughout the service’s lifecycle. This extended timeframe allows for an evaluation of both initial adoption patterns and subsequent changes that might be masked in cross-sectional or shorter-term studies. By tracking key variables, such as user ratings, sentiment polarity, and service modifications over time, the study offers a more robust assessment of how digital-first models can rise, plateau, or decline in response to both internal operations and external circumstances.

### Data sources and data analysis

A total of 1,308 online user reviews from NHS Reviews (69), Trustpilot (616), and Google Reviews (623) were collected between 2017 and 2024. These included numerical ratings (1–5 stars) and 1,128 textual comments. User feedback provides insight into patient experiences, satisfaction levels, and perceived service quality. Newspaper articles, online media, and policy briefings were also analysed to understand the broader societal and policy discourse, offering context for changes in service perception and adoption. Peer-reviewed studies, reports, and guidelines on digital-first healthcare models provided additional evidence on broader trends and challenges relevant to eMed GP at Hand.

#### Sentiment analysis

TextBlob [11] and NVivo [12] were employed for sentiment analysis of the textual reviews. TextBlob (Python library) quantifies sentiment polarity (ranging from − 1 for negative to + 1 for positive) and subjectivity (0 for objective to 1 for highly subjective). Its ease of use and language-processing capabilities make it suitable for a large volume of short-form text such as user reviews. NVivo (qualitative data analysis software) complements TextBlob’s numerical scoring with a more nuanced, qualitative coding approach. NVivo supports manual and automated text categorization, enabling deeper thematic exploration and corroborating the automated sentiment scores.

Alternative sentiment analysis tools were considered (e.g., VADER, Stanford CoreNLP, or Google Cloud Natural Language) which offer different algorithms and lexicons. For instance, VADER is optimized for social media text, while Stanford CoreNLP can provide more detailed linguistic parsing. TextBlob was chosen for its balance between simplicity and accuracy in handling general English reviews, and NVivo for its robust qualitative coding features. Using two tools helped validate sentiment findings through both automated scoring and human-in-the-loop exploration, reducing the risk of biases in any single method.

#### Thematic analysis

A thematic analysis [13] was then conducted to interpret the qualitative feedback from user reviews and media sources. This approach enabled the identification of recurring patterns (e.g., convenience, continuity of care, accessibility) and provided qualitative depth to the sentiment findings. Themes that emerged from NVivo coding were refined through an iterative process, resulting in seven major themes detailed in the Findings section.

### Theoretical lens and hypothesis

To interpret the trajectory of eMed GP at Hand, the study applies the Gartner Hype Cycle [14] as a theoretical framework for understanding technology adoption within healthcare. The Hype Cycle posits that new technologies move through stages of initial hype, inflated expectations, disillusionment, eventual enlightenment, and a potential plateau of productivity. Based on this model, the research posits the following hypothesis:

#### Hypothesis

As eMed GP at Hand progresses from initial enthusiasm (‘peak of inflated expectations’) to potential disillusionment (‘trough of disillusionment’), user satisfaction and media sentiment will exhibit a corresponding rise, subsequent dip, and potential stabilization over time. By mapping user ratings, media coverage, and policy stances onto the Hype Cycle’s phases, the study aims to test whether the observed patterns in adoption and satisfaction align with the model’s theoretical progression.

The directed content analysis was guided by constructs derived from the Gartner Hype Cycle, which informed the categorisation of media narratives and user discourse according to stages of technological expectation and disillusionment.

## Findings

### Analysis of eMed GP at hand user ratings and reviews

1,308 users of GP at Hand left ratings ranging from 1 to 5 stars. As shown in Table 2 and 66.51% of users gave 4- or 5-star ratings, indicating generally positive feedback, while 30.2% gave 1- or 2-star ratings, reflecting notable dissatisfaction with the service.

**Table 2:** Count of reviews from 1 to 5 as proportion of total

Rating	Total count	Proportion of total
1	326	24.92%
2	69	5.28%
3	43	3.29%
4	75	5.73%
5	795	60.78%

**Fig. 1:**
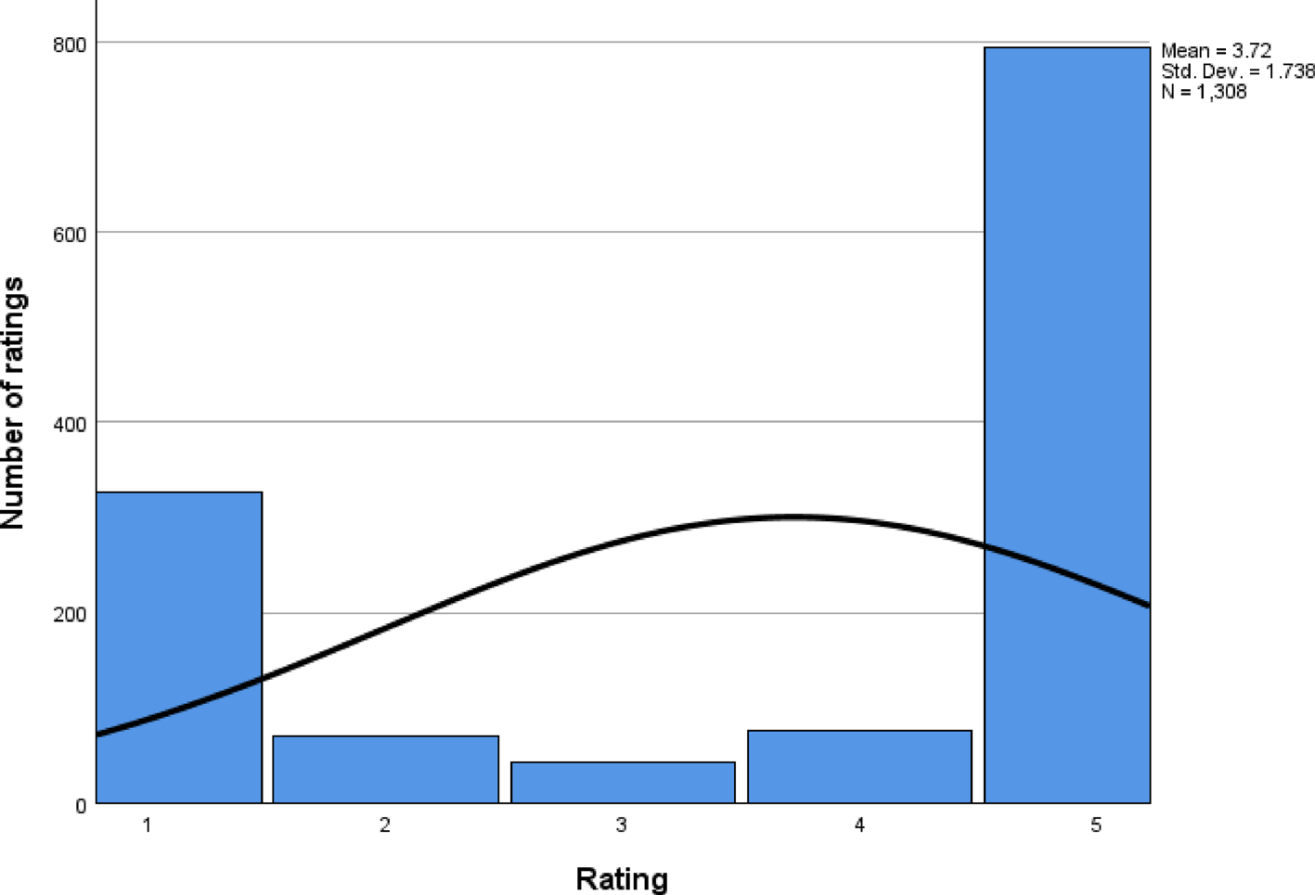
Graph showing the total number of ratings along with the distribution curve

The average rating was 3.77, with a mean of 3.72 (σ = 1.74), suggesting overall positive feedback. The median and mode were both 5, indicating a strong positive skew, while the standard deviation of 1.74 and variance of 3.02 showed significant variability. The left-skewed distribution (skewness = −0.77) pulled the mean below the median, while a kurtosis of −1.27 suggested a relatively uniform spread, with ratings concentrated at the higher end. Figure 1 illustrates two distinct user experience patterns: a majority of highly satisfied users and a smaller but significant group of dissatisfied users. The low frequency of 2-, 3-, and 4-star ratings indicates polarised opinions, with most users expressing either strong approval or disapproval.

The quartile analysis emphasises the polarised distribution of ratings. The 25th percentile (Q1) was 2, indicating that 25% of users rated the service 2 or lower, whilst the median and 75th percentile (Q3) were both 5, showing that half of all users gave the maximum rating. This clustering of high and low ratings highlights a dichotomy in user experiences, with a significant proportion of the ratings clustered at the extremes (Table 3).

**Table 3:** Table showing the percentiles for the user ratings

Percentiles								
		5	10	25	50	75	90	95
Weighted average	Rating	1.00	1.00	2.00	5.00	5.00	5.00	5.00
Tukey’s Hinges	Rating			2.00	5.00	5.00		

**Fig. 2:**
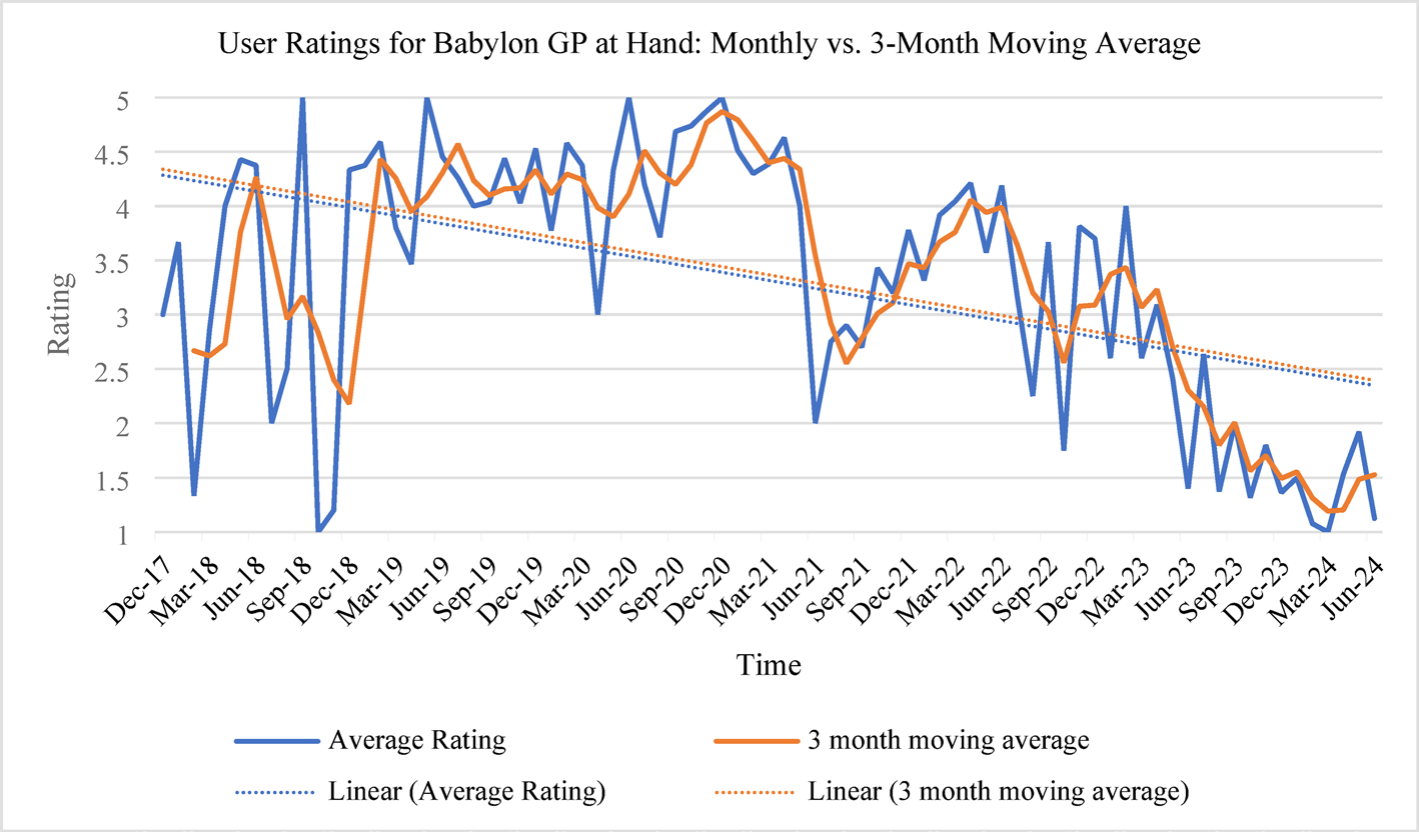
Comparison of Monthly Average Ratings and 3-Month Moving Average Ratings for Babylon GP at Hand (Dec 2017 – June 2024)

**Fig. 3:**
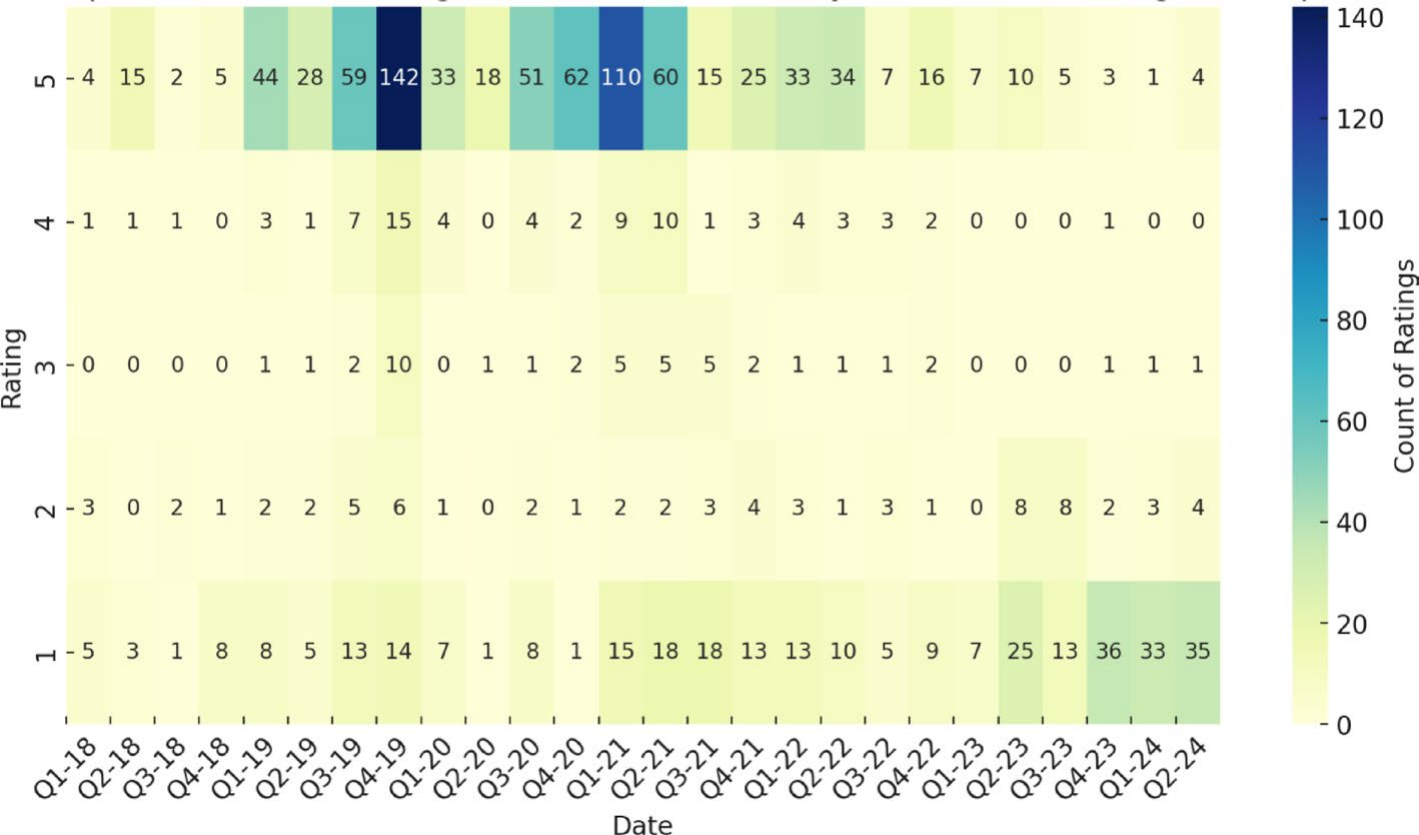
Heatmap of customer ratings and count over time

Graphs of average (Fig. 2) and heatmap of ratings over time (Fig. 3), along with a 3-month moving average, reveal a decline in user satisfaction over time, marked by downward trendlines. The inclusion of the 3-month moving average smooths short-term fluctuations, providing a clearer view of the overall trend while mitigating the impact of isolated spikes or dips in ratings. Fluctuations in ratings could reflect service updates, changing user expectations, or external factors, such as shifts in healthcare demand. The service initially performed well during the pandemic, with high ratings due to heightened demand and reliance on remote care. However, as restrictions lifted in mid-2021, ratings declined, suggesting difficulties in maintaining quality amid the shift back to in-person services. The 3-month moving average further highlights this sustained decline, reinforcing the trend rather than attributing it to isolated monthly variations. Following the transition from Babylon GP at Hand to eMed GP at Hand, average ratings dropped from 4.05 to 2.46, indicating decreased user satisfaction, potentially due to changes in service quality, user experience, or policy shifts.

The descriptive statistics reveal a complex satisfaction profile. While many users reported high satisfaction, a significant minority had poor experiences, indicating systemic issues warranting further investigation. The left-skewed and platykurtic distribution suggests the need for a more detailed analysis of user feedback to capture the diverse range of experiences.

#### Textual reviews

Analysing the 1,128 textual reviews alongside the ratings provides a deeper understanding of user satisfaction. While numerical ratings offer a quick snapshot, they lack the context and detail needed to fully capture user experiences. Textual reviews provide valuable insights into specific issues, emotional sentiments, and both positive and negative aspects of the service. By examining these reviews, it becomes possible to uncover the reasons behind the ratings, allowing for a more comprehensive assessment of user satisfaction and the overall service quality.

#### Sentiment analysis

Sentiment analysis was conducted using the TextBlob library in Python, calculating two metrics: sentiment polarity (emotional tone, ranging from − 1 to 1) and subjectivity (degree of opinion, scaled from 0 to 1), metrics shown in Table 4. This approach enables a nuanced understanding of feedback, distinguishing between objective statements and subjective, emotionally driven opinions.

**Table 4:** Table of descriptive statistics for sentiment analysis

	Sentiment Polarity	Subjectivity
Count	1128	1128
Mean	0.24	0.62
Standard Deviation	0.43	0.17
Minimum	−1.0	0.1
25%	−0.18	0.52
50%	0.39	0.61
75%	0.56	0.72
Maximum	1.0	1.0

The analysis showed a mean sentiment polarity of 0.24 (σ = 0.43), indicating mildly positive overall feedback, which aligns with the mean rating of 3.72. The median polarity was 0.39, skewed towards positive sentiment, while the 25th percentile (−0.18) reflected negative reviews, and the 75th percentile (0.56) highlighted highly positive feedback. These metrics suggest varied user opinions, with a general trend towards positive sentiment, though less polarised than the numerical ratings.

Subjectivity analysis revealed that reviews were predominantly opinion-based, with an average score of 0.62 (σ = 0.17), indicating a high level of personal experience in the feedback. The median subjectivity score of 0.61 closely matched the mean, showing a balanced distribution. The 25th percentile (0.52) indicated less subjective reviews, while the 75th percentile (0.72) showed most reviews leaned towards higher subjectivity, reflecting the personal nature of healthcare experiences.

**Fig. 4:**
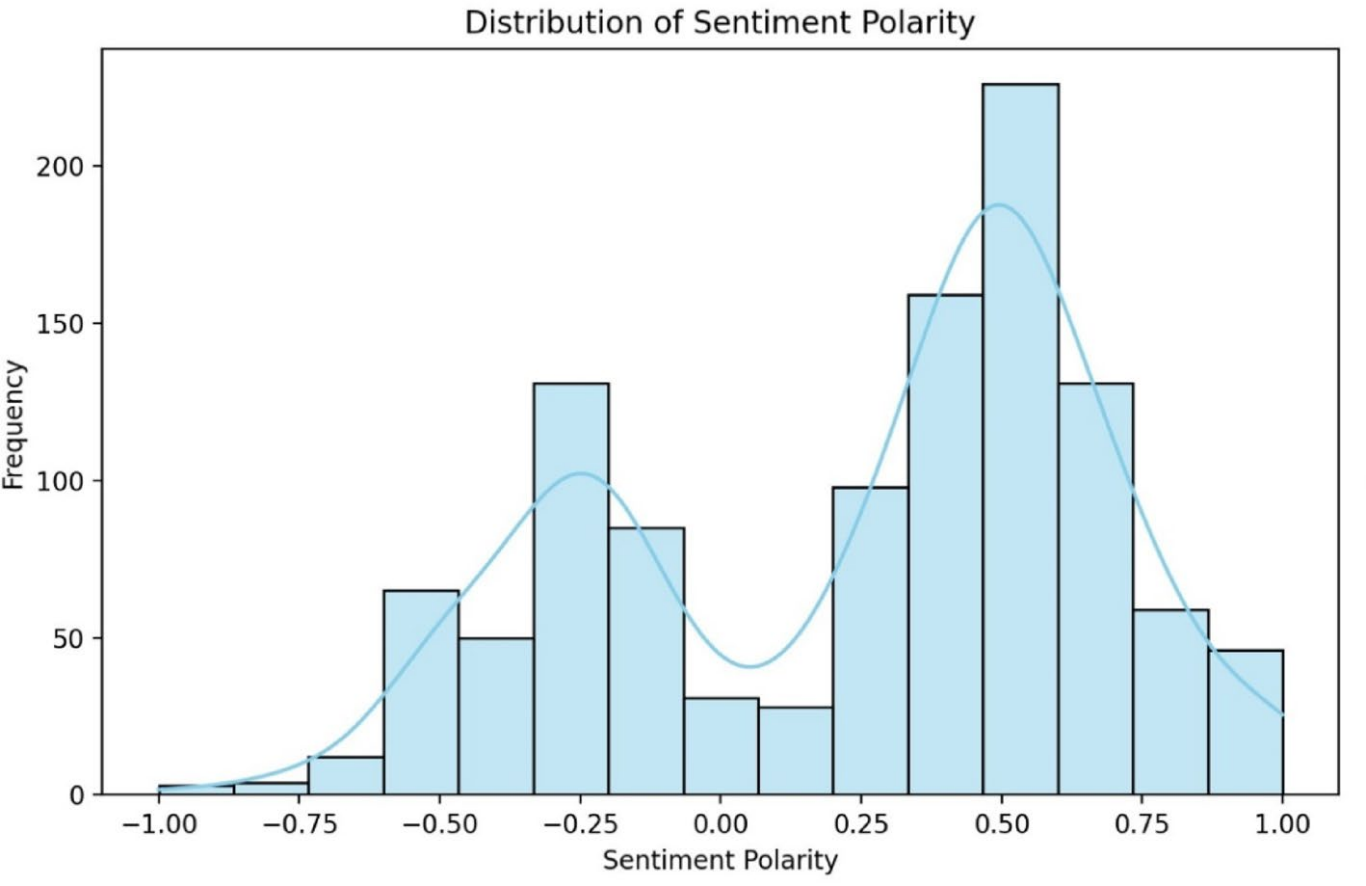
Histogram of sentiment polarity distribution for eMed GP at Hand reviews

The histogram of sentiment polarity (Fig. 4) reveals a slightly skewed distribution towards positive values, with a peak in the mild positive range, consistent with the summary statistics indicating generally positive feedback. The right tail extends toward maximum positive sentiment, capturing highly positive reviews, while the left tail, though less pronounced, reflects negative sentiment. This diversity highlights the range of user experiences. The kernel density estimate (KDE) overlay confirms a tilt towards positive sentiment, indicating that most reviews leaned favourably despite notable dissatisfaction.

Sentiment trends were also influenced by external events like the COVID-19 pandemic and policy changes. Early in the pandemic, sentiment polarity scores frequently exceeded 0.3, reflecting appreciation for the convenience of digital healthcare during lockdowns. However, sentiment declined in late 2020 and early 2021, with scores dropping to around 0.05, driven by frustrations with service limitations, technical issues, and the strain of prolonged isolation. Negative reviews often cited bureaucratic inefficiencies and difficulties with the healthcare system. Sentiment improved during the vaccine rollout, as optimism grew, although concerns about service access and vaccination procedures still contributed to negative feedback. These trends illustrate the dynamic relationship between service delivery, external challenges, and user satisfaction.

**Fig. 5:**
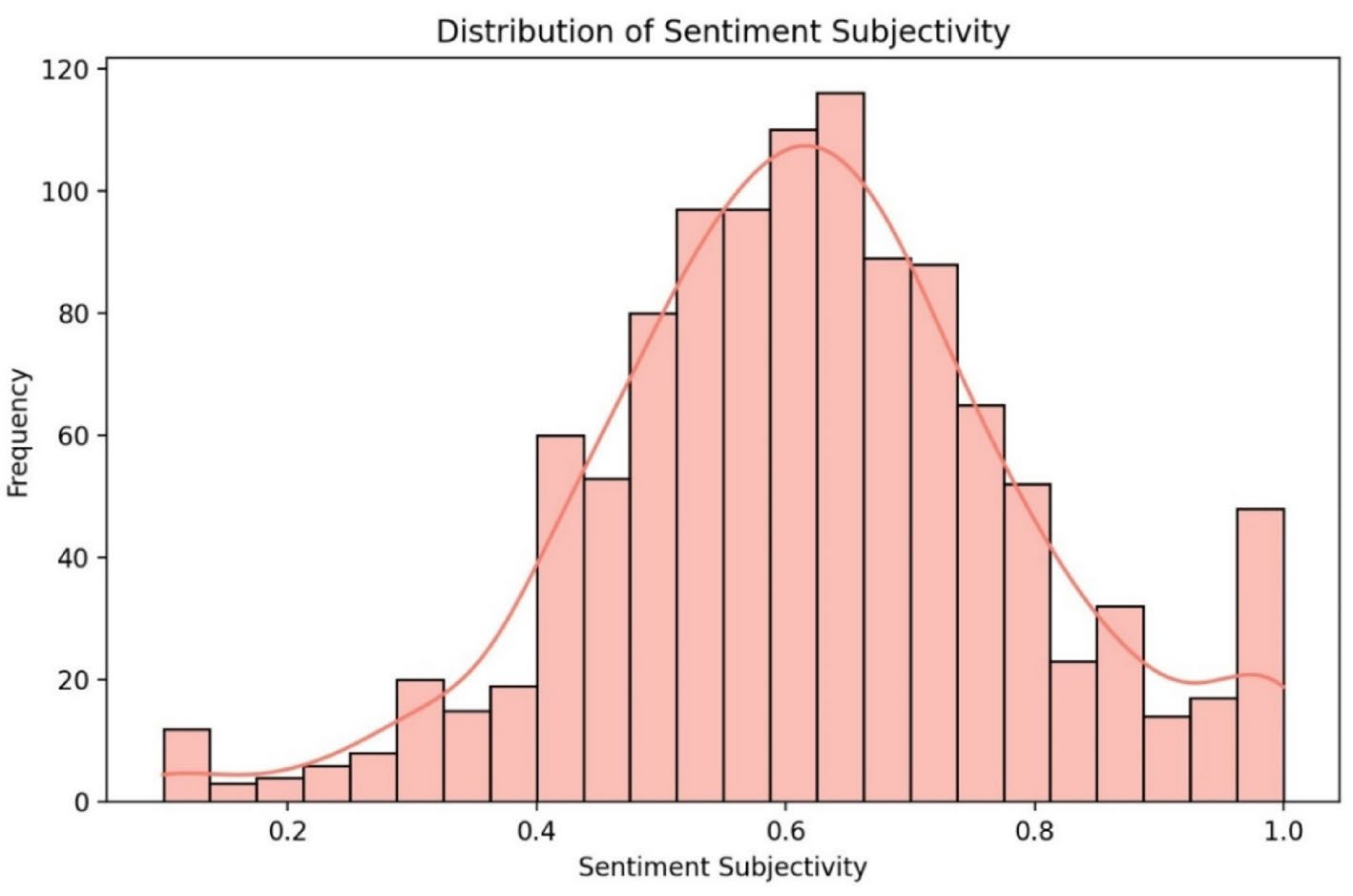
Histogram of subjectivity distribution for eMed GP at Hand reviews

The histogram of subjectivity (Fig. 5) shows a more symmetrical spread compared to sentiment polarity, with a slight right skew, indicating that reviews often consist of personal opinions rather than purely factual accounts. The central peak around the mean subjectivity score of 0.62 suggests moderate to high subjectivity in many reviews. The range, from 0 (objective) to 1 (highly subjective), captures diverse review styles, from fact-based feedback to emotional, opinion-driven narratives. The KDE overlay confirms the tendency towards subjective experiences, complementing the sentiment analysis and providing insight into the emotional tone of the feedback.

#### Sentiment examples

Examples from the data reveal a range of user experiences. Positive reviews often cite the service’s convenience, such as: “Perfect for busy lives - I was seen by a doctor (via the app) within an hour of booking an appointment…prescription was sent to the chemist next to my work instantly.” This review, with a sentiment polarity of 0.28 and a subjectivity score of 0.49, balances factual content and opinion, consistent with its 5-star rating. Conversely, negative reviews reflect frustrations, as in: “It was supposed to make the whole process easier and more convenient…there are so many unnecessary rules… I don’t understand why I could get medication from my former GP but now GP at Hand told me they are unable to prescribe it due to their regulation. What kind of regulation is that? Not prescript the medication the patients need? Then what’s the point of the GP? Total waste of time and energy,” This review has a sentiment polarity of −0.5 and a subjectivity score of 0.7, indicating strong negative sentiment and aligning with the 1-star rating.

The juxtaposition of numerical ratings and sentiment scores highlights an interesting contrast. While ratings show polarisation, sentiment analysis reveals a more nuanced range of opinions. This divergence may be due to several factors: textual reviews can express mixed emotions and subtleties that ratings cannot, limitations of the 5-point scale may push users towards extreme ratings, and cognitive dissonance may cause users to give extreme ratings even when their written sentiment is more moderate.

**Fig. 6:**
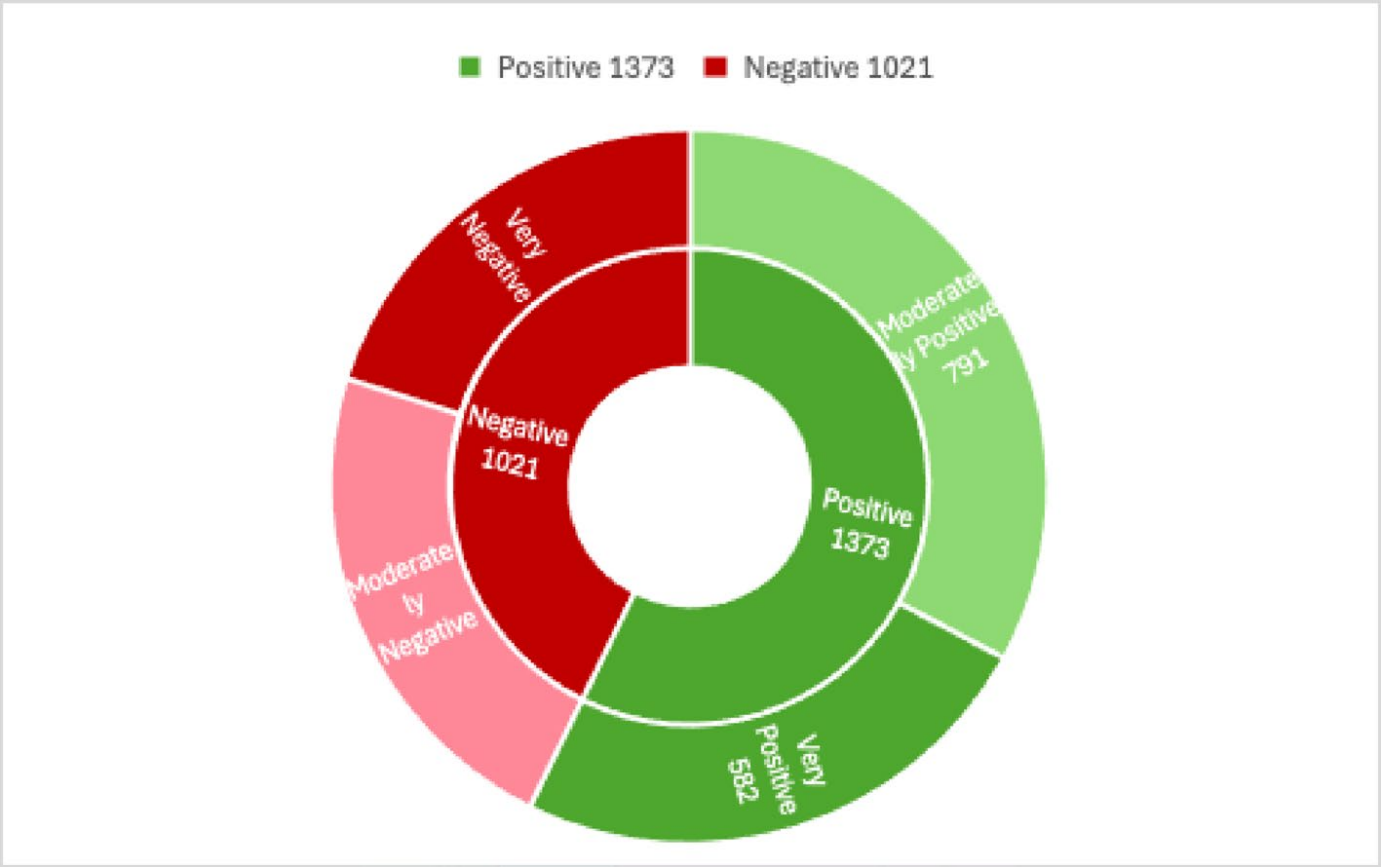
Sunburst diagram of sentiment references in the eMed GP at Hand reviews

To enhance sentiment analysis validity, NVivo categorized the 1,128 reviews into “Very Positive,” “Moderately Positive,” “Very Negative,” and “Moderately Negative.” Of the 2,394 sentiment references, 57.35% (1,373) were positive and 42.65% (1,021) were negative. Within these, “Moderately Positive” sentiments made up 57.61% of positive references, while “Moderately Negative” sentiments comprised 52.69% of negative ones, reflecting a nuanced spectrum of feedback (see Fig. 6).

The consistency between NVivo’s qualitative categorization and TextBlob’s automated sentiment scoring supports the robustness of the sentiment patterns identified, bolstering the credibility of the findings. The slight skew towards positive sentiment across both methods suggests that user feedback genuinely reflects satisfaction with eMed GP at Hand. However, the moderate level of subjectivity in reviews indicates a blend of opinions and facts, offering valuable insights into both strengths and improvement areas for the service. This dual-method approach, combining quantitative and qualitative analyses, provides a comprehensive understanding of user experiences, informing strategies to enhance user satisfaction and address key concerns.

#### Word cloud

**Fig. 7:**
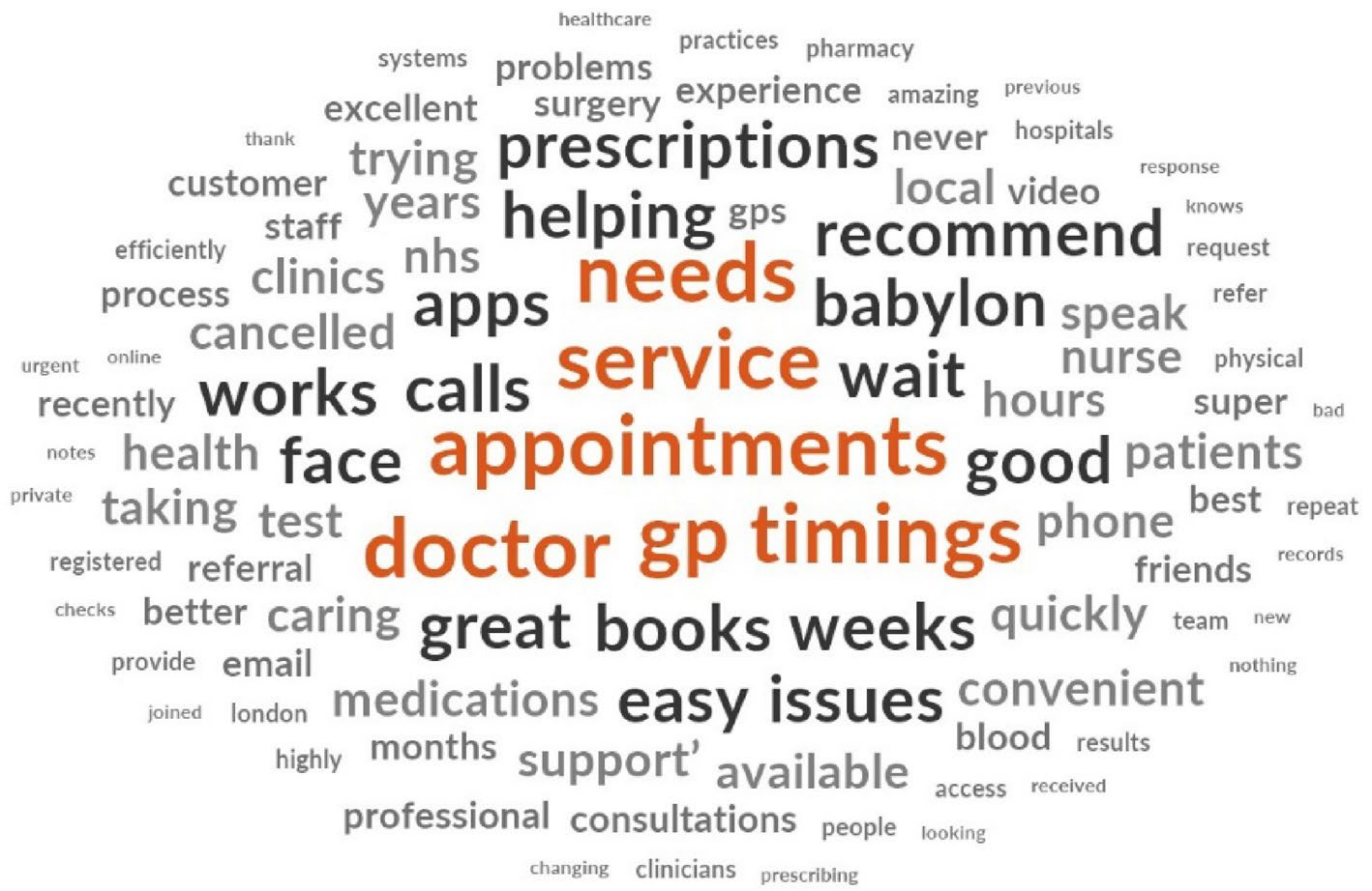
Word cloud visualisation created using the eMed GP at Hand reviews

Prior to thematic analysis, a word cloud was generated using NVivo to visualize word frequency in the eMed GP at Hand reviews (see Fig. 7). The visualization shows key themes, with larger words indicating higher occurrence. Frequent terms such as “appointments,” “service,” “doctor,” “gp,” and “needs” suggest a strong user focus on scheduling, service quality, and interactions with medical professionals. Positive descriptors like “easy,” “quickly,” “great,” and “good” indicate user satisfaction with efficiency, aligning with the earlier sentiment analysis that skewed towards positive feedback.

Operational aspects are highlighted by terms like “timings,” “calls,” “book,” and “online,” emphasizing logistical concerns around scheduling and communication. Healthcare-specific terms, including “medications,” “health,” “nhs,” and “clinics,” reflect the core medical functions such as prescription management and NHS integration. Technological terms, such as “apps” and “online,” underscore the digital-first nature of the service, while words related to user experience, like “customer,” “support,” and “helping,” indicate the importance of user interaction and support. This word cloud analysis offers a high-level overview of the key topics and sentiments expressed in the reviews, setting the stage for a deeper thematic exploration of user experiences and perceptions in the case study.

#### Thematic analysis

Thematic analysis of the user reviews, guided by Braun and Clarke’s [13] framework, identified seven major themes, each offering distinct insights into users’ perceptions and experiences:

- Convenience and accessibility
- Quality of care
- Administrative and technical Issues
- Prescription and referral management
- Comparison to traditional GP practices
- Patient satisfaction and recommendations
- Service evolution
  A. *Convenience and accessibility*

Convenience was the most prominent theme, with many users praising the service for fitting seamlessly into busy lifestyles, especially for minor health issues. Reviews highlighted the ease of digital access, with a user describing the service as “perfect for busy lives“(Anonymous, 8 December 2017), whilst another said, “perfect when you have an inflexible working schedule,” (Anonymous, 14 March 2018) due to short-notice availability and video consultations outside regular hours.

However, not all users experienced this convenience. Those outside London reported geographic limitations, with one Yorkshire resident finding the service unsuitable: “Not for me though” (Anonymous, 18 December 2017). In London, some users criticized the scarcity of clinics and aftercare options: “It’s practically impossible to get aftercare unless you live locally to one of these clinics” (Anonymous, 26 February 2018). These limitations contradicted the service’s promise of broad accessibility and may have influenced eMed GP at Hand’s exit from Birmingham in October 2022, which faced criticism from healthcare professionals and resistance from the Hammersmith and Fulham CCG.

B. *Quality of care*

Quality of care emerged as a central topic, with users often expressing satisfaction with appointment speed and doctor competence, contrasting it with traditional GP wait times. For example, one user found the service “a million times better than going to my old doctors” (Anonymous, 30 September 2019). Users also valued the ability to access consultation notes digitally, which enhanced convenience, as one user wrote, “consultation notes were conveniently saved after the conversation,” (Anonymous, 4 February 2019).

However, variability in care quality across consultations was a common concern. One user noted, “The quality of service between GPs varies massively” (Anonymous, 22 August 2019), while another commented, “appalling…Practitioners are a hit or miss - some are very good, and some are mediocre " (Anonymous, 3 April 2023). This inconsistency highlights a challenge in digital-first models: maintaining a consistent standard of care. Additionally, the lack of continuity was problematic, as patients could not consistently consult the same doctor, leading to fragmented care for ongoing health concerns. This contrasts with traditional GP practices, where ongoing relationships with the same GP allow for tailored, continuous care. One review emphasized this issue, stating, “it’s hard to see the same GP twice since they have 1000 s … feels more like a conveyer belt, so end up getting wildly different opinions on what to do " (Anonymous, 12 April 2024).

The theme highlights the inherent tension between the convenience of digital-first and the traditional expectations of continuity and consistent quality. While eMed GP at Hand excels in providing quick access, it struggles to deliver consistent quality and continuity. Continuity is crucial for managing ongoing health needs and a lack of it potentially risks patient safety and satisfaction due to fragmented care across a rotating pool of doctors.

C. *Administrative and technical issues*

Users frequently cited administrative and technical problems that impacted their experience. Common issues included app malfunctions, difficulties with ID verification, poor coordination between technical support and clinical teams, and unresponsive customer support. One user reported, “The app won’t verify my ID… reception stated they do have my ID and don’t know why the app isn’t working for me” (Anonymous, 8 December 2017). Another user highlighted long wait times for call centre assistance when a technical issue prevented follow-up appointment booking, stating, “Technical support is virtually non-existent… I spend literally hours trying to reach their call line” (Anonymous, 28 May 2021). Such challenges undermined the service’s perceived reliability, prompting frustration, reduced trust and in some cases leading to users abandoning the service. The frequency of these complaints indicates that robust technical infrastructure and user interface are critical for effective digital healthcare delivery.

D. *Prescription and referral management*

Feedback on prescription and referral management was mixed. Some users praised the speed of receiving prescriptions, such as, “Prescription sent electronically to my nearest pharmacy within 10 minutes” (Anonymous, 21 March 2019). However, others reported recurring issues with prescriptions not arriving at pharmacies and delays in specialist referrals, requiring extensive follow-up. For example, one review noted, “Prescriptions don’t arrive at the pharmacies… it’s just shambles” (Anonymous, 27 January 2022). Referral delays, sometimes lasting several weeks, exacerbated frustrations, especially for urgent cases. These gaps highlight the need for refining prescription and referral processes to ensure smooth service delivery.

E. *Comparison to traditional GP practices*

Users frequently compared eMed GP at Hand to traditional GP services, often emphasizing advantages like accessibility and convenience. Positive feedback noted the ease of securing appointments outside regular hours and accessing medical records through the app: “You can speak to a GP at 3 am… the treatment plan is also saved in the app” (Anonymous, 3 June 2020). Users appreciated the quality of service compared to overcrowded traditional practices, as one noted, “Much better service via the app than at my overcrowded GP” (Anonymous, 20 December 2019).

However, limitations surfaced when dealing with chronic or complex conditions. For instance, one user described difficulties accessing mental health services due to geographical restrictions, feeling trapped in a “Catch-22”: “you cannot be referred via Community Mental Health because GP at Hand isn’t in your local Borough - and you cannot be referred via NHS Right To Choose because GP at Hand only has a contract with them for ADHD Assessments - and not ASD” (Anonymous, 16 November 2022). Additionally, users expressed concerns about the lack of support during emergencies, which often shifted the burden onto other NHS services: “Absolutely no way to speak to someone in an emergency… I have had to call 111 unnecessarily” (Anonymous, 24 April 2024).

This theme emphasises the debate about the scope and limitations of digital healthcare services. While eMed GP at Hand excels in providing accessible and convenient care, it struggles with managing chronic or complex conditions requiring continuity and comprehensive coordination with local services. Thus, the service is better suited as a supplement to, rather than a replacement for, traditional GP practices, especially for patients needing comprehensive or continuous care.

F. *Patient satisfaction and recommendations*

User satisfaction was notably polarised, with some users expressing high levels of satisfaction and recommending the service enthusiastically. A user praised the service, in relation to their healthcare system in their home country, saying, “coming from a country without national health care, this is a revelation. This needs to go global” (Anonymous, 6 February 2019). One user even described the service as “the best thing since sliced bread” (Anonymous, 18 May 2023).

Conversely, dissatisfaction was prevalent among users encountering technical problems or complex health needs. One user expressed regret after facing administrative challenges, saying, “At first this seemed like the perfect solution… I am leaving this service for a traditional GP, one that cares about its patients” (Anonymous, 13 March 2019). Another user, frustrated with technical issues, commented, “I’m literally held hostage to an obvious back-end bug… For once, a traditional GP might actually be better” (Anonymous, 3 August 2021). The polarised feedback indicates that the effectiveness of digital-first models depends heavily on the nature of a patient’s healthcare needs and may not be suitable for everyone, particularly those with more complex medical conditions.

G. *Service evolution*

The analysis revealed a dynamic evolution of the service, marked by significant early highs and recent lows. Users praised the innovative model, describing it as “the future of medicine” (Anonymous, 11 February 2021), and questioned the need for conventional GPs: “I honestly cannot understand why anyone would sign up to a conventional GP anymore” (Anonymous, 22 March 2021).

However, recent reviews indicate a decline in quality, particularly after the transition to eMed. Users reported longer wait times and a perceived drop in care standards, with some expressing disappointment over the service’s trajectory since the takeover: “eMed have run it into the ground” (Anonymous, 7 January 2024). Others criticized the shift toward corporate interests, stating, “shame on Emed for destroying a great service and shame on Babylon for selling out to these corporate vultures” (Anonymous, 14 June 2024). The decline in average ratings over time underscores a crucial lesson in digital health service evolution; innovation can quickly attract users and generate enthusiasm, but sustaining high levels of service quality and addressing user concerns is essential for long-term satisfaction.

### Media and academic literature

When eMed GP at Hand first launched, it rapidly became one of the largest and fastest-growing general practices in England, attracting considerable attention from policymakers, clinicians, and the media. While patients and staff expressed satisfaction, the broader impact on the healthcare system prompted calls for rigorous research into the implications of digital-first [16]. Although then Health Secretary Matt Hancock endorsed the app enthusiastically, some argued that this support prioritized enthusiasm over necessary caution, with concerns about the lack of robust evidence for its efficacy [17]. Critics highlighted the absence of evidence showing improvements in patient outcomes and NHS efficiency, as well as potential risks to patient safety and resource allocation.

The service also faced criticism for “cherry-picking” younger, healthier patients, as GP at Hand primarily attracted individuals who lived or worked within 40 min of the practice. According to the Ipsos MORI evaluation, GP at Hand’s user base tended to be younger, healthier, and more affluent than the general population. This pattern disadvantaged traditional practices, which were left with older patients who had more complex health needs but received reduced funding due to the Carr-Hill Formula. The CCG (Hammersmith and Fulham) responsible for Babylon struggled financially and required NHS intervention to reallocate millions in funding [18]. Additionally, vulnerable populations without internet access were also at risk of being excluded from such digital-first care models.

The service’s flexible, remote GP workforce facilitated same-day appointments but created a sense of disconnect from patients, contrasting with traditional community-based care. Accessibility issues for older adults, individuals with complex needs, and those with limited digital skills raised concerns about exacerbating health inequalities. High deregistration rates, with 28% of users leaving within two weeks, highlighted dissatisfaction, particularly with in-person care. Questions also emerged about whether GP at Hand was addressing unmet healthcare needs or generating supply-induced demand [9].

A central issue was Babylon’s AI-driven symptom-checking chatbot, which faced criticism for diagnostic inaccuracies. A 2018 Forbes report cited Babylon’s own medical staff expressing doubts about the AI’s reliability [19]. While Babylon emphasized its commitment to safety, scepticism persisted, with a former employee claiming that marketing was prioritized over clinical validation, and clinical trials were avoided due to their cost and complexity [18].

Regulatory concerns arose when the Medicines and Healthcare products Regulatory Agency (MHRA) received complaints from clinicians about the chatbot’s failure to identify serious illnesses. This raised critical questions about the effectiveness and safety of the technology [20, 22]. Oncologist Dr David Watkins, a longstanding critic of Babylon, tested the app’s accuracy and found that it often failed to recognize critical health conditions. He highlighted disparities in how the chatbot triaged heart attack symptoms in male versus female patients, misdiagnosing the latter with anxiety or depression [22]. Watkins argued that Babylon’s claims that the app was “100% safe” and as effective as a human doctor, arguing that such assertions were irresponsible and posed potential risks to patients [23]. Watkins also documented other issues, such as misleading terms and conditions, exaggerated AI capability claims, governance concerns, and a lack of mechanisms to track patient outcomes post-triage. He suggested that these flaws could hinder the identification of adverse events [20].

The MHRA’s regulatory response was also criticized. Watkins argued that the regulatory framework at the time was inadequate for overseeing AI-driven health technologies, leaving the sector largely self-regulated [20]. The MHRA acknowledged that the regulatory framework for software-based medical devices was insufficient, particularly for AI, and that comprehensive updates might take years to implement, leaving a gap in oversight and accountability for emerging technologies [20].

Media reporting at the time characterised Babylon’s response to clinical criticism as adversarial, raising concerns about the organisation’s approach to professional and regulatory challenge [23]. Other critics, such as Dr Margaret McCartney, a GP from Scotland, also raised concerns with regulatory bodies, including the MHRA, Care Quality Commission (CQC), Advertising Standards Authority, and the Royal College of General Practitioners (RCGP). However, Babylon’s financial connections to supportive politicians and lack of transparent evidence delayed meaningful regulatory intervention [24].

In 2020, Babylon faced further scrutiny when a user discovered a data breach that allowed access to recorded video consultations of other patients on the app. A subsequent investigation by Babylon revealed that a small number of additional UK users could also view others’ sessions and attributed the breach to a ‘small’ software error [25], downplaying its significance. However, the incident raised serious concerns about patient data security and the overall integrity of digital-first healthcare models.

Concerns about the model’s suitability for more complex patient groups persisted, despite some positive findings in the Ipsos MORI evaluation [16]. Research has shown continuity of care improves health outcomes and reduces unplanned hospital admissions, a factor that is especially critical for older patients with multiple health conditions. While the digital-first model improved healthcare access for some demographics, it inadequately supported vulnerable populations, particularly the elderly and those with limited digital skills. The Centre for Ageing Better reported that 25% of individuals aged 65 + lacked internet access at home, and over 40% faced limitations in online activities [26]. Similarly, Age UK indicated that more than a third of people over 65 (around 4.7 million people) lacked basic internet skills, with one in six not using the internet at all. An elderly patient shared concerns with Age UK, stating, “It is very difficult to get a medical appointment now and my surgery is pushing more and more services online. It has got to the point where access is so difficult, I don’t seek advice and just hope minor conditions just go away” [27].

Despite objections from Birmingham and Solihull CCG regarding the service’s expansion, eMed GP at Hand grew to become the largest practice in England. However, financial pressures led to the closure of its Birmingham operations in 2022, followed by Babylon’s administration in August 2023. The UK operations were subsequently acquired by eMed, limiting services to the London area [21, 28, 29].

Responding to criticism about “cherry-picking” younger, tech-savvy patients, Dr Tim Rideout, president of global clinical services, stated that the service did not actively exclude any demographic but naturally attracted a working-age adult population [30]. However, this response did not address the digital divide or propose solutions for inclusivity. It also overlooked the public health implications of a model inadvertently contributing to health inequities. Rideout acknowledged the failed expansion, admitting the service was not adequately connected with referral and integrated care networks [30].

Dr Jay Verma, a London GP, noted that younger patients frequently presented with mental health concerns, which Babylon’s model did not fully accommodate. NHS data shows that younger people, particularly women aged 17–25, are more likely to seek help for mental health issues [31]. While user reviews praised the mental health support provided, the funding model (Carr-Hill formula) generated less revenue from younger patients, leading to financial unsustainability. Instead of fostering long-term relationships, Babylon’s approach was transactional, focusing on short-term gains without building a sustainable care model [32].

The model did facilitate streamlined appointment allocation by enabling online triage, reducing reliance on a “first-come, first-served” approach. Nevertheless, Verma argued that the business model’s failure to address the complexities of patient populations contributed to its downfall. He contended that healthcare models should prioritise patient needs over demand, which Babylon’s promise of “anytime, anywhere” access failed to achieve.

Despite setbacks, eMed GP at Hand remains England’s largest practice [30], reflecting significant demand for digital-first healthcare. However, its success in Hammersmith and Fulham, a borough with a high proportion of young, working-age residents (23% aged 25–34) [33], may not be generalizable across England. The demographic differences between Hammersmith and Fulham and broader populations (see Table 5) suggest that the model may not meet the needs of more diverse patient groups effectively.

**Table 5:** Population percentage by age group in Hammersmith and Fulham, compared to London and England

Age Group	% of Hammersmith and Fulham Population	Higher or lower (compared to London & England)
17 and under	19%	Lower
25–34	23%	Higher
50–64	15%	Lower
65 and above	10%	Lower

The demographic profile of Hammersmith and Fulham is key to understanding eMed GP at Hand’s success, driven by the needs of younger, healthier individuals facing difficulties accessing traditional GP services. For instance, young adults unable to get appointments or take time off work may find the service’s convenience appealing for minor ailments. However, older patients with complex conditions requiring continuity of care may find it inadequate, potentially worsening health inequalities by failing to provide sustained and relational care. The service’s withdrawal from Birmingham underscored that success in one demographic or geographic context does not ensure scalability elsewhere.

Moreover, assuming that younger individuals are healthier and thus less reliant on GP services oversimplifies the unpredictability of health. Patient reviews suggest that the model may not meet the needs of those with sudden or serious health issues, highlighting its design focus on minor, episodic needs rather than evolving healthcare priorities. Consequently, the model struggles with sustainability in delivering comprehensive general practice, as it lacks adaptability to the dynamic nature of healthcare.

### Comparison to the Gartner hype cycle

**Fig. 8:**
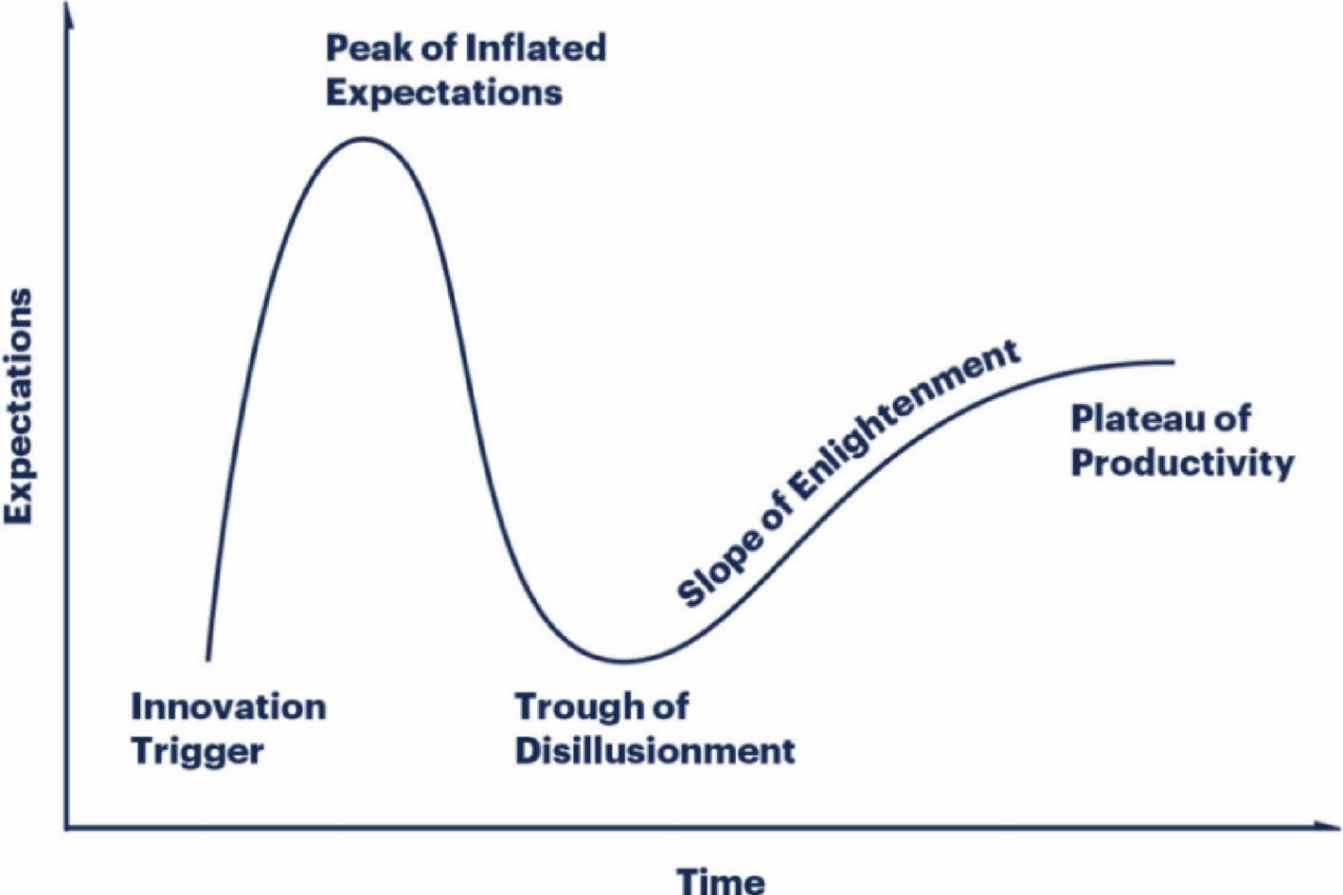
The Gartner Hype Cycle [15]

The Gartner Hype Cycle outlines five stages in the evolution of technologies: Innovation Trigger, Peak of Inflated Expectations, Trough of Disillusionment, Slope of Enlightenment, and Plateau of Productivity (see Fig. 8). Each stage reflects shifts in public expectations and the technology’s performance, serving as a strategic tool for decisions related to emerging technologies, resource allocation, and investment planning [15].

Applied to eMed GP at Hand, the Hype Cycle provides insights into its development and impact on user satisfaction and trust. The Innovation Trigger marks the initial excitement surrounding a breakthrough, despite uncertainties about commercial viability, leading to the Peak of Inflated Expectations. Here, early successes and setbacks generate both enthusiasm and scepticism among stakeholders. As hype wanes, the technology enters the Trough of Disillusionment, where challenges and implementation issues cause interest to decline, and some organisations may abandon investment. In the Slope of Enlightenment, successful applications emerge, benefits are clearer, and refinements address earlier issues. The Plateau of Productivity represents mainstream adoption, with the technology demonstrating clear market value [15].

The pace of progression through these phases varies. Some technologies linger in specific stages, affecting their perceived value, while others quickly reach the Plateau of Productivity. Not all technologies complete the cycle; some may be abandoned, while others oscillate between phases, evolving and reshaping their evaluation [15].

The eMed GP at Hand case study aligns with distinct phases of the Gartner Hype Cycle, highlighting key trends in its technological life cycle:

#### Innovation trigger

The Innovation Trigger phase began with Babylon Health’s announcement that its AI diagnostic chatbot outperformed human doctors in a medical exam, scoring 81% compared to 72% for human doctors [34]. This generated significant excitement, positioning Babylon as a leader in AI healthcare. Public interest was further fuelled by endorsements from Health Secretary Matt Hancock, who publicly supported and used the service, and extensive media coverage, amplifying expectations. However, enthusiasm was based more on anticipated breakthroughs than on proven, scalable solutions, leading to inflated expectations.

#### Peak of inflated expectations

Between 2019 and 2020, the Peak of Inflated Expectations coincided with growing interest in telemedicine, amplified by the COVID-19 pandemic. The pandemic and pre-existing NHS pressures, such as routine GP appointment wait times exceeding two weeks in 2019 [35], accelerated the adoption of digital health services like eMed GP at Hand. Rising demand increased optimism about digital health solutions. While users praised the service’s convenience and its potential to revolutionise healthcare, early adopters encountered typical ‘teething issues,’ reflected in fluctuating ratings and varied user experiences. This phase was marked by optimism but also highlighted the growing pains associated with new technologies. For example, during the pandemic, numerous self-triage tools were rapidly deployed to support overwhelmed health systems. However, as Ziebart et al. [36] note, many of these tools lacked integration with clinical systems and were insufficiently evaluated for usability or effectiveness, raising concerns about their actual utility in reducing healthcare demand.

#### Trough of disillusionment

From late 2021 onward, eMed GP at Hand entered the Trough of Disillusionment, reflected in declining user ratings. Several factors contributed to this downturn:

- User Disillusionment – As adoption grew, systemic issues like long wait times, technical difficulties, and unmet expectations became more evident. Users expressed dissatisfaction, with many citing the service as “oversubscribed” and no longer “convenient.”
- Market Saturation – The post-pandemic surge in telehealth services introduced more competition, shifting user expectations and leading to declining satisfaction as users compared options.
- Regulatory Changes – Post-pandemic, the NHS implemented stricter guidelines to address concerns over potential health inequalities exacerbated by the platform and its impact on traditional primary care services. This led to a pause in their expansion, the closure of its Birmingham operations, and a halt to out-of-area registrations. Babylon’s sale to eMed amid financial struggles further eroded user confidence, as reflected in negative reviews.

These factors collectively contributed to perceptions of the service’s unsustainability, driving further declines in user satisfaction.

#### Slope of enlightenment and plateau of productivity

eMed GP at Hand currently remains in the Trough of Disillusionment, with its transition to the Slope of Enlightenment or Plateau of Productivity uncertain. Progress will depend on stabilising user satisfaction, rebuilding trust, and aligning service capabilities with user needs. This may occur as user expectations adjust and the service demonstrates consistent performance. However, the recent acquisition by eMed has further reduced user satisfaction, suggesting that mainstream acceptance and long-term viability may take time.

## Discussion

**Fig. 9:**
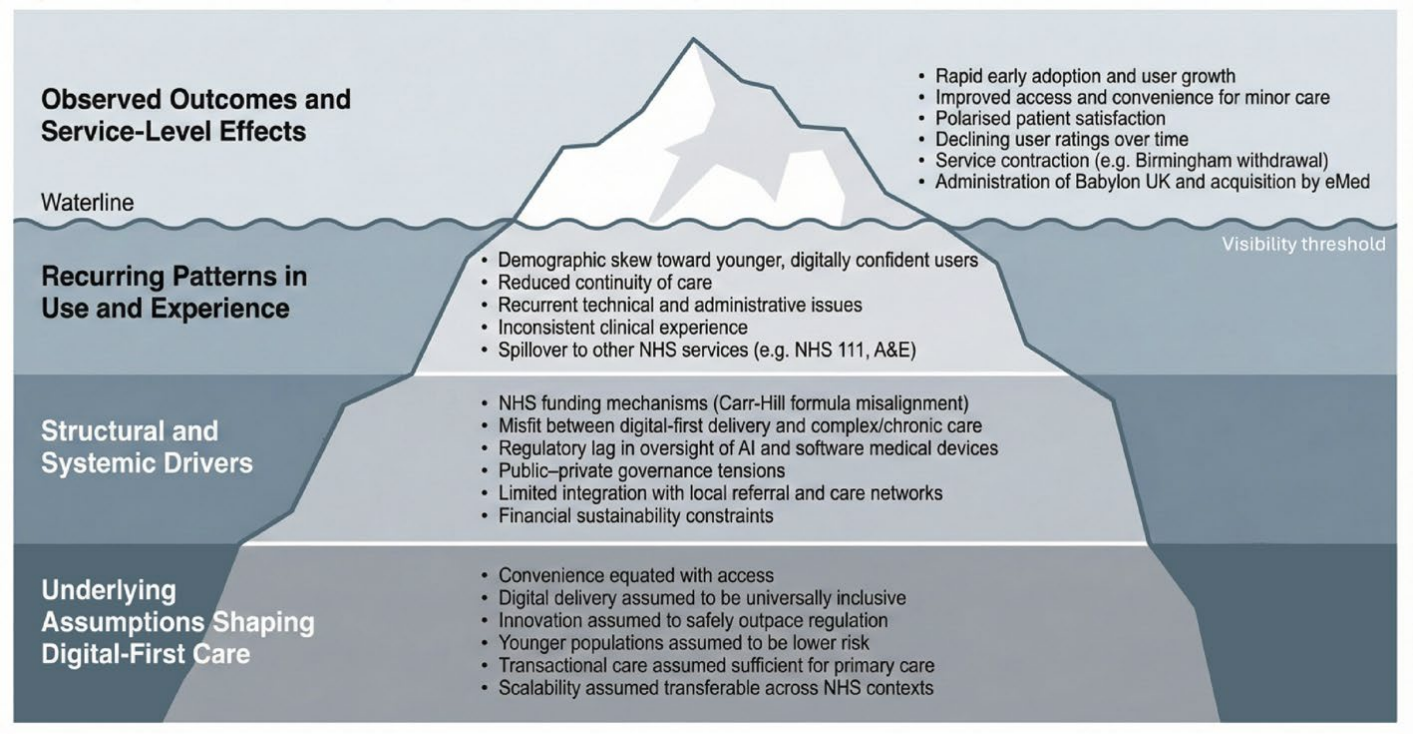
Systemic Factors Shaping Digital-First Primary Care Outcomes in the NHS

To synthesise the discussion of systemic influences on digital-first primary care, Fig. 9 presents an iceberg model illustrating how visible service-level outcomes are shaped by deeper structural drivers and underlying assumptions.

Despite its innovative model, GP at Hand has been controversial [37, 38], with criticisms targeting the reliability of its AI chatbot, the limited scope of its services, and its funding approach. NHS England initially blocked its expansion pending the independent Ipsos MORI evaluation but lifted the ban before the findings were released, sparking governance concerns [16, 18, 39]. The evaluation revealed a user base skewed towards younger, affluent individuals − 75% aged 20–34 - who valued rapid access to GP consultations. Digital consultations, particularly for mental health, received positive feedback, while face-to-face services faced criticism due to limited availability and administrative inefficiencies [16, 18, 39]. Whilst the evaluation offered a snapshot in time assessment, this research expands on those findings by offering insights into changing user experiences and the broader implications in an evolving healthcare landscape.

The thematic analysis of user reviews reveals both strengths and limitations, reflecting the complexities inherent in a digital-first healthcare model. Initially, the service was praised for its convenience, accessibility, and efficiency, particularly for routine medical issues. Users valued the quick access to care and the digital interface’s ease of use. However, as the service expanded, challenges emerged in appointment availability, technical reliability, continuity of care, and scalability.

A trend of dissatisfaction is evident, especially concerning the service’s ability to manage complex and ongoing healthcare needs. Users reported inconsistencies in clinical interactions, communication problems, and difficulties in providing continuity of care for chronic conditions. Additionally, disparities between the free and paid versions of the app, with more available appointments for paying users, fuelled concerns about a bias toward private healthcare. This perceived further contributed to user dissatisfaction, leading some to express regret for joining the service and intentions to “leave” or “de-register” and return to traditional GP practices.

As demand grew, criticisms regarding inefficiencies, oversubscription, and poor customer support increased, indicating that the service struggled to scale effectively. This led to a decline in service quality and user trust, as evidenced by falling ratings over time. While the digital interface remains a valued component of the service, these challenges have eroded the early success of the service. Addressing issues such as care continuity, technical reliability, and equitable access will be essential for the service to regain and sustain user confidence as it continues to evolve.

The regulatory gap raises important questions about accountability in digital health. If the oversight mechanisms for AI-driven medical technologies remain underdeveloped, who is ultimately responsible for ensuring their safety and effectiveness before they reach the public? The case of Babylon illustrates how companies can make bold claims about their products despite an evolving regulatory landscape. This misalignment between innovation and regulation not only introduces potential risks to patient safety but also challenges the role of regulatory bodies in protecting the public. Without clear and enforceable standards, there is a risk that commercial interests may outpace patient safeguards, highlighting the need for a more responsive and adaptive regulatory framework. Despite widespread promotion of digital-first services, a significant evidence gap remains. As noted by the NIHR’s rapid evidence synthesis [40], much of the existing research is based on perceptions or technology features, with very limited objective outcome data comparing digital models to standard primary care. This includes a lack of robust evidence on health outcomes, diagnostic accuracy, cost-effectiveness, or continuity of care.

The 2020 data breach raised serious concerns about patient data security and the overall integrity of digital-first healthcare models. Patient health data is highly sensitive, and even minor system errors can result in significant privacy breaches, undermining public trust. More broadly, this breach exemplifies a fundamental challenge in digital-first healthcare: the reliability and security of technological infrastructures. The increasing reliance on AI-driven and digital platforms necessitates rigorous testing and validation to ensure they function as intended under all conditions. In this context, the ability of Babylon’s app to safeguard patient data should have been a central consideration in its design and implementation. The breach raises critical questions about the adequacy of Babylon’s testing protocols and whether they sufficiently accounted for potential security vulnerabilities. If digital-first healthcare is to be a viable alternative to traditional models, robust safeguards must be in place to prevent such incidents and maintain patient confidence in these technologies.

Whilst the Ipsos evaluation lacked an investigative angle into continuity of care, the current research, specifically the analysis of the user reviews, has shown that lack of continuity is a significant issue experienced by users, one that cannot be sustained by the digital-first service. In traditional GP practices, patients often form relationships with their GP, enabling a tailored and continuous care pathway. However, in the eMed model where patients are frequently matched with different doctors, this consistency is compromised. The user reviews accentuate the inherent tension between the convenience of digital-first care and the traditional expectations of continuity and consistent quality in healthcare. This fragmentation of care poses risks to patient safety and satisfaction, as important details may be overlooked or inconsistently addressed across multiple consultations.

This emphasises that the digital divide disproportionately affects older individuals, which was warned of in the early days of Babylon. There is a risk that the benefits of digital healthcare could be unevenly distributed across the population, leaving vulnerable groups underserved and potentially worsening health disparities. Recent research reinforces these findings. Shapiro et al. [41] propose a multi-domain framework for enterprise-level digital health adoption that places equity on par with usability, interoperability, and workflow integration. Their approach underscores that digital solutions must be assessed not only for technical performance but also for their capacity to serve diverse patient populations, particularly those who are traditionally underserved or face barriers to digital access.

Additionally, Babylon patients frequently faced longer travel times for in-person appointments compared to traditional GP practices. While Ipsos MORI indicated that most patients were aware of this trade-off when registering, it remained unclear how those with mobility issues or requiring frequent in-person care would manage [16]. Though whilst not an explicit answer, user reviews revealed that some patients resorted to visiting A&E for issues typically managed by GPs, suggesting a shift in the burden of care to other parts of the healthcare system.

These setbacks raise questions about the long-term sustainability of digital-first models under NHS funding constraints, reflecting broader challenges faced by private companies attempting to innovate within the public healthcare system.

## Recommendations and conclusion

Several recommendations for digital health development emerge. First, investing in robust, user-friendly technical infrastructure is vital to maintaining patient trust and service reliability. Second, ensuring consistent quality across digital and traditional interactions is necessary to provide comparable care regardless of access medium. Integrating digital services into existing healthcare systems is essential to prevent fragmentation and support continuity of care. This integration is vital for delivering seamless patient experiences and ensuring that digital platforms complement, rather than replace, traditional healthcare services. Additionally, tailored digital solutions for patients with chronic or complex conditions are needed to address diverse healthcare requirements. Scalability efforts must prioritise quality care as user numbers increase, and continuous user feedback analysis will be crucial for refining services to complement traditional healthcare.

While services like GP at Hand offer significant benefits in accessibility and convenience for minor health issues, they struggle with managing complex conditions and ensuring continuity of care. The lack of consistent, relational care poses risks to patient safety, emphasizing the need for high-quality, continuous care. Administrative and technical challenges further highlight the importance of a seamless user experience. Comparisons with traditional GP practices show that while GP at Hand is valued for convenience, it often falls short in addressing complex healthcare needs, stressing the need for digital services to complement rather than replace traditional care.

Babylon’s recent collapse serves as a cautionary tale, illustrating the complexities of integrating private sector innovation into public healthcare. The withdrawal from NHS contracts raises critical questions about the sustainability of private sector involvement, particularly when financial and profit-driven motives conflict with the NHS’s goal of delivering equitable and patient-centred care. The limitations of Babylon’s AI, which struggled with serious condition diagnoses, underscore the risks associated with deploying unproven technologies at scale. These shortcomings stress the need for careful planning, strong regulation, and a comprehensive understanding of patient needs to ensure that digital healthcare models can deliver sustainable and equitable care.

Dr Watkins’ critique and the MHRA’s response reveal significant gaps in regulatory oversight with implications for patient safety. The tension between rapid technological innovation and the slower pace of regulatory adaptation threatens public trust in digital health solutions. The MHRA’s admission of insufficient regulatory frameworks for digital technologies highlights systemic regulatory issues. The reliance on company-driven safety validation, rather than independent and rigorous testing, creates a conflict of interest that distorts public perceptions of these technologies’ safety and efficacy.

Babylon’s promotional practices appear to have been part of a broader strategy to inflate market value at the expense of transparency and patient safety. The ongoing use of misleading claims, even after regulatory interventions, suggests a persistent disregard for ethical marketing practices. From a governance perspective, Babylon prioritized expansion and investor relations over addressing safety concerns, revealing a disconnect between patient expectations and the actual service delivery. This misalignment is a critical area for improvement if digital primary care models are to serve a broader, more diverse patient population effectively. A ‘one-size-fits-all’ approach is unsuitable in the complex, varied, and ever-changing landscape of primary care.

In conclusion, this case study highlights the challenges of regulating AI and digital healthcare in a rapidly evolving landscape. As illustrated in Fig. 9, these challenges are not solely technical but reflect deeper structural and governance constraints. Findings call for urgent development of regulatory frameworks that keep pace with technological advancements while prioritizing patient safety. Ethical concerns raised by Babylon’s practices serve as a warning to health tech companies navigating the balance between innovation, governance, and public trust. Whilst Dr Watkins’ experience serves as a reminder of the importance of fostering an environment where concerns can be raised without fear of retribution or public discreditation. The need for continuous improvement in digital health services, particularly in addressing complex patient needs, is evident. As digital health evolves, striking the balance between innovation and accountability will be crucial to ensure these technologies realize their potential without compromising patient safety.

## Data Availability

A dataset of consumer review comments was compiled from disparate online sources in the public domain. This compiled set was analysed during the current study, and is available from the corresponding author on reasonable request.
